# Internalizing problems before and during the COVID-19 pandemic in independent samples of Dutch children and adolescents with and without pre-existing mental health problems

**DOI:** 10.1007/s00787-022-01991-y

**Published:** 2022-05-26

**Authors:** Karen Fischer, Jacintha M. Tieskens, Michiel A. J. Luijten, Josjan Zijlmans, Hedy A. van Oers, Rowdy de Groot, Daniël van der Doelen, Hanneke van Ewijk, Helen Klip, Rikkert M. van der Lans, Ronald De Meyer, Malindi van der Mheen, Maud M. van Muilekom, I. Hyun Ruisch, Lorynn Teela, Germie van den Berg, Hilgo Bruining, Rachel van der Rijken, Jan Buitelaar, Pieter J. Hoekstra, Ramón Lindauer, Kim J. Oostrom, Wouter Staal, Robert Vermeiren, Ronald Cornet, Lotte Haverman, Meike Bartels, Tinca J. C. Polderman, Arne Popma

**Affiliations:** 1 Department of Biological Psychology, Vrije Universiteit Amsterdam, Amsterdam, The Netherlands; 2 Amsterdam University Medical Centres, Amsterdam Public Health Research Institute, Amsterdam, The Netherlands; 3 LUMC Curium–Child and Adolescent Psychiatry, Leiden University Medical Center, Leiden, The Netherlands; 4 Emma Children’s Hospital, Amsterdam University Medical Center, Child and Adolescent Psychiatry and Psychosocial Care, Amsterdam Reproduction and Development, University of Amsterdam, Amsterdam, The Netherlands; 5 Amsterdam University Medical Center, Epidemiology and Data Science, Vrije Universiteit Amsterdam, Amsterdam, The Netherlands; 6 Department of Child and Adolescent Psychiatry and Psychosocial Care, Amsterdam University Medical Center, Vrije Universiteit Amsterdam, Amsterdam, The Netherlands; 7 Department of Medical Informatics, Amsterdam University Medical Center, University of Amsterdam, Amsterdam, The Netherlands; 8 Karakter Child and Adolescent Psychiatry University Centre, Nijmegen, The Netherlands; 9 Praktikon, Nijmegen, The Netherlands; 10 Levvel, Academic Center for Child and Adolescent Psychiatry, Amsterdam, The Netherlands; 11 Department of Child and Adolescent Psychiatry, Amsterdam University Medical Center, University of Amsterdam, Amsterdam, The Netherlands; 12 Department of Child and Adolescent Psychiatry, University Medical Center Groningen, University of Groningen, Groningen, The Netherlands; 13 Netherlands Youth Institute, Utrecht, The Netherlands; 14 Department of Cognitive Neuroscience, Donders Institute for Brain, Cognition and Behaviour, Radboudumc, Nijmegen, The Netherlands; 15 Youz, Parnassia Psychiatric Institute, den Hague, The Netherlands

**Keywords:** Internalizing problems, Mental health, COVID-19, Anxiety, Depression, Children and adolescents, Coronavirus

## Abstract

The aim of the study was to assess internalizing problems before and during the pandemic with data from Dutch consortium Child and adolescent mental health and wellbeing in times of the COVID-19 pandemic, consisting of two Dutch general population samples (GS) and two clinical samples (CS) referred to youth/psychiatric care. Measures of internalizing problems were obtained from ongoing data collections pre-pandemic (*N*_GS_ = 35,357; *N*_CS_ = 4487) and twice during the pandemic, in Apr–May 2020 (*N*_GS_ = 3938; clinical: *N*_CS_ = 1008) and in Nov–Dec 2020 (*N*_GS_ = 1489; *N*_CS_ = 1536), in children and adolescents (8–18 years) with parent (Brief Problem Monitor) and/or child reports (Patient-Reported Outcomes Measurement Information System^®^). Results show that, in the general population, internalizing problems were higher during the first peak of the pandemic compared to pre-pandemic based on both child and parent reports. Yet, over the course of the pandemic, on both child and parent reports, similar or lower levels of internalizing problems were observed. Children in the clinical population reported more internalizing symptoms over the course of the pandemic while parents did not report differences in internalizing symptoms from pre-pandemic to the first peak of the pandemic nor over the course of the pandemic. Overall, the findings indicate that children and adolescents of both the general and clinical population were affected negatively by the pandemic in terms of their internalizing problems. Attention is therefore warranted to investigate long-term effects and to monitor if internalizing problems return to pre-pandemic levels or if they remain elevated post-pandemic.

**Supplementary Information:**

The online version contains supplementary material available at 10.1007/s00787-022-01991-y.

## Introduction

**Fig. 1:**
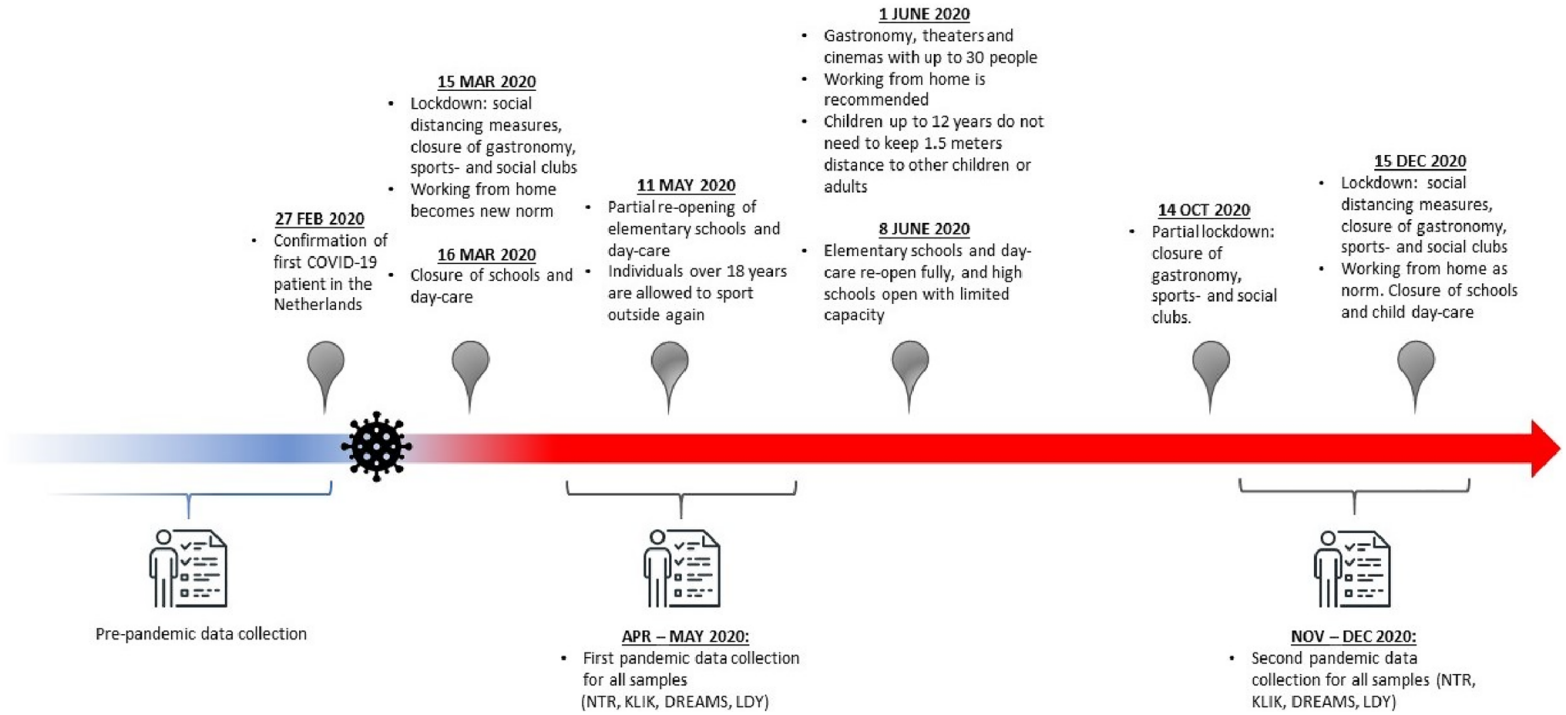
Timeline COVID-19 Regulations in the Netherlands

The implemented social distancing measures during the COVID-19 pandemic have brought about marked changes in the daily lives of people across the globe. Restrictions, such as primarily working at home, closure of schools and limited physical contact with friends and family, have characterized life during the COVID-19 pandemic lockdown (see Fig. 1 for a detailed description of the restrictions over time in The Netherlands). The effects of the restrictions are especially of concern regarding the psychosocial development of children and adolescents, since social interactions and forming relationships with peers—which were both limited during the COVID-19 pandemic—are crucial components of a healthy development during this age [1]. Social deprivation may contribute to feelings of loneliness, disconnection from one’s peers, and experiencing internalizing problems like depressive and anxious feelings [2]. In addition, the fear of the virus itself and the uncertainty of how this might affect one’s family or the world in general may negatively affect children’s and adolescents’ mental health [3]. A large body of literatures show that uncontrollable events with a potentially large impact, can have long-lasting negative consequences on mental health, in particular on the development of anxiety and depressive symptoms [4, 5]. Therefore, it is important to gain insight into levels of internalizing symptoms in children and adolescents during the current pandemic.

Several cross-sectional studies from China conducted in children and adolescents in the general population [6–8] indicated higher prevalence of anxiety and depressive symptoms during the first lockdown than pre-pandemic; however, these differences were not statistically assessed. Initial results from one of our general population-based samples [9] are in line with these findings, showing that children and adolescents (*N* = 844) reported more anxiety and depressive symptoms during the first COVID-19 lockdown in the Netherlands (Apr. 2020), compared to a reference sample before the pandemic. Similarly, another general population-based study in Germany (*N* = 1556), also using a reference sample as pre-pandemic measure, found that two-thirds of children reported more mental health problems and a decline in health-related quality of life since lockdown began [10]. Longitudinal studies up to this date corroborate this pattern. For example, a study from the UK (*N* = 168) showed that children (aged 7–11) reported an increase in depressive symptoms during the first lockdown, when compared to their ratings 18 months earlier before the pandemic, and that this effect did not differ across age, gender, and family socioeconomic status (SES) [11]. Another longitudinal study in 248 adolescents showed that self-reported depressive and anxiety symptoms were higher two months into the pandemic than in the year preceding the pandemic [12]. In addition, a longitudinal study in children and adolescents (aged 9–18 years) from the US, The Netherlands and Peru (*N* = 1339), showed an increase in depressive symptoms from pre-pandemic to the first half year of the pandemic [13]. As these studies were conducted exclusively in the general population, it remains less clear how the pandemic affects children’s internalizing problems in vulnerable groups, such as those with pre-existing mental health problems. Initial findings from our group [14] showed that during the pandemic children in psychiatric care self-reported more depressive symptoms, but not more anxiety than children from the general population. A recent systematic review on the effects of the pandemic on adolescent mental health shows that adolescents with pre-existing mental health conditions experienced a worsening in their pre-existing conditions with onset of the pandemic [15].

In light of this literature, studies using larger and more diverse samples—ranging from general to referred clinical populations—are necessary to yield a clearer picture regarding variations and divergence in mental health in children and adolescents before and during the pandemic. To gain such insights, we investigated the effects of the COVID-19 pandemic on internalizing problems in children and adolescents between 8 and 18 years with and without pre-existing mental health problems in four separate cohorts: two large Dutch general population-based cohorts and two Dutch clinical cohorts. Specifically, we assessed child and parent reports on internalizing problems before the pandemic and at two measurements during the pandemic in independent samples (between-subjects design) to investigate whether levels of internalizing symptoms, as well as proportions of children with heightened internalizing problems, differ before and over the course of the pandemic.

## Methods

### Participants

Data were used from children and adolescents of 8–18 years from the Dutch consortium *Child and adolescent mental health and wellbeing in times of the COVID-19 pandemic*, which is a unique Dutch collaboration consisting of four large child and adolescent cohorts: two general population-based ongoing cohorts (the Netherlands Twin Register (NTR) and KLIK), and two clinical cohorts (Dutch Research in child and Adolescent Mental health (DREAMS) and Learning Database Youth (LDY)). Below we will provide a short description of the different cohorts and Table 1 gives an overview of the sample characteristics per cohort. An extensive description of the separate cohorts and respective details of data collection procedures can be found in the supplementary materials.

**Table 1:** Sociodemographic information of the samples before and during the pandemic for all cohorts

Cohort	Pre-pandemic				Pandemic Apr–May 2020				Pandemic Nov–Dec 2020			
	*N*	%Males	%Mother ratings	*M*_Age_ (SD)	*N*	%Males	%Mother ratings	*M*_Age_ (SD)	*N*	%Males	%Mother ratings	*M*_Age_ (SD)
General population												
Parent-reported internalizing problems (BPM)												
NTR	34,038	49.5%	97.7%	10.4 (1.9)	3524	53.7%	84.7%	9. 2 (2.1)	1168	49.0%	88.1%	9.8 (2.3)
Child-reported internalizing problems (PROMIS)												
KLIK	1319	49.4%	N/A^a^	12.7 (3.1)	832	46.3%	N/A^a^	13.4 (2.8)	746	53.3%	N/A^a^	13.7 (3.2)
Clinical population												
Parent-reported internalizing problems (BPM)												
DREAMS	1395	61.9%	N/A^b^	11.4 (2.4)	453	55.6%	79.9%	13.1 (3.1)	726	58.5%	84.4%	13.2 (2.9)
LDY	3092	62.3%	68.8%	13.2 (3.0)	280	62.5%	61.4%	13.4 (2.9)	302	64.6%	53.0%	13.7 (3.0)
Child-reported internalizing problems (PROMIS)												
DREAMS					275	54.2%	N/A^a^	13.0 (3.1)	508	50.2%	N/A^a^	13.9 (2.9)

*BPM *Brief problem monitor, *PROMIS P*atient-reported outcomes measurement information system

^a^Not applicable due to child report measure

^b^Not applicable due to unknown informant

### Cohorts of the general population

#### NTR

The NTR [16] was established in 1987 and collects data at multiple times during development in twins and multiples from birth onwards. The pre-pandemic measurement includes data from 1995 up to 2019 resulting in a sample of 34,038 children (49.5% boys). The first pandemic measurement (Apr–May 2020) consisted of 3,524 children (53.7% boys). The second pandemic measurement (Nov–Dec 2020) consisted of 1168 children (49.0% boys).

#### KLIK

Data in this cohort were collected through a research website (www.hetklikt.nu) of the KLIK Patient-Reported Outcome Measures (PROM) portal developed specifically for this purpose (www.corona-studie.nl). The samples are representative of the Dutch general population [21]. The pre-pandemic measurement consisted of 1,319 children (49.4% boys) collected in 2018 [21]. The first pandemic measurement (April 2020) consisted of 832 children (46.3% boys). The second pandemic measurement (Nov 2020) consisted of 746 children (53.3% boys).

### Cohorts of the clinical population

#### DREAMS

DREAMS is a collaboration between four academic child and adolescent psychiatric centers in the Netherlands (Amsterdam, Groningen, Leiden, Nijmegen) together covering the northern, western, and eastern part of the Netherlands. All children receiving psychiatric care and their parents were invited to participate by e-mail through their psychiatric center. As with the KLIK sample, data were collected through a research website. For parent reports, the pre-pandemic measurement consisted of 1395 children (61.9% boys). The first pandemic measurement (Apr–May 2020) consisted of 453 children (55.6% boys). The second pandemic measurement (Nov–Dec 2020) consisted of 726 children (58.5% boys). For the child reports, the first pandemic measurement (Apr–May 2020) consisted of 275 children (54.2% boys). The second pandemic measurement (Nov–Dec 2020) consisted of 508 children (50.2% boys).

#### LDY

LDY is a cooperation between youth care centers in the Netherlands to collect data on the mental health status of children and adolescents who receive youth care, to improve quality of care. In this study, data of 14 youth care institutions were used. The youth care centers are situated in northern (12.7%), eastern (60.8%), southern (2.2%) and western (24.3%) parts of the Netherlands. Participating children and adolescents in the LDY sample receive youth care for various problems, such as mental, pedagogical, or educational problems. Data collection was part of their treatment trajectory, where caregivers were asked to fill out questionnaires before, during, and at the end of treatment. The pre-pandemic sample (Jan–Dec 2019) consisted of 3,092 children (62.3% boys). The first pandemic measurement (Apr–May 2020) consisted of 280 children (62.5% boys). The second pandemic measurement (Nov–Dec 2020) consisted of 302 children (64.6% boys).

## Design and procedure

Parent and/or child reports on internalizing problems were collected once before the pandemic and twice during the pandemic in independent samples over time within the four different cohorts. For the DREAMS cohort, no pre-pandemic child-reported data were available. Pre-pandemic measures were obtained from ongoing data collections that took place at various time points before the pandemic. These data were collected anywhere between 2018 and 2019, with the exception that for NTR the pre-pandemic assessments reached back to 1995. Data at the first pandemic measurement were collected in Apr–May 2020, during the first peak of the pandemic when there was a strict lockdown in The Netherlands. Data at the second pandemic measurement were collected in Nov–Dec 2020, when there was a partial lockdown (schools reopened) in the Netherlands. See Fig. 1 for a timeline of the most important regulations that were active in the Netherlands at the time of our data collection. Prior to the start of the study, collaborating parties received approval for data collection by the appropriate ethics committees, and all children and parents provided informed consent. Data from the LDY sample were not collected specifically for this study but as part of patients’ treatment trajectory. The studies were conducted in line with the ethical standards stated in the 1964 Declaration of Helsinki and its later amendments.

## Measures

### Parent-reported internalizing problems

#### Brief problem monitor (BPM)

The BPM [18] is a shortened version of the Child Behavior Checklist-6–18 years; [19]), which is a widely used questionnaire on behavioral- and emotional problems in children. To assess internalizing problems, the internalizing problem scale was used, consisting of 6 items about anxious, withdrawn and depressed symptoms. Items were rated on a three-point Likert scale, reflecting how much a statement applies to their child (0 = ‘not true’, 1 = ‘somewhat true’, to 2 = ‘very true’). Internal consistency of the internalizing subscale is (*α*) = 0.80 [18]. In line with the BPM manual, missing items were coded as zero [18] and reports were excluded if more than 20% of the responses to the items within the scale were missing. Item scores were summed to yield a total score.

### Child-reported internalizing problems

#### Patient-reported outcomes measurement information system (PROMIS^®^)

The Dutch–Flemish PROMIS^®^ (Patient-Reported Outcomes Measurement Information System) pediatric V2.0. Item Bank Anxiety and V2.0. Item Bank Depressive Symptoms were used to assess child-reported internalizing problems and are developed using modern psychometric techniques [20] that measure their respective domains of anxiety and depressive symptoms in children. The Anxiety and Depressive Symptoms [21] item banks were administered as Computerized Adaptive Tests (CAT), where items are selected based on responses to previously completed items, resulting in a reliable score with a few items. The anxiety item bank contains 15 items that reflect fear (e.g., fearfulness), anxious misery (e.g., worry), and hyperarousal (e.g., nervousness) [21]. The depressive symptoms item bank contains 14 items on negative mood (e.g., sadness), anhedonia (e.g., loss of interest), negative views of the self (e.g., worthlessness, low self-esteem), and negative social cognition (e.g., loneliness, interpersonal alienation) [21]. All PROMIS measures use a 7 day recall period, and most items are scored on a five-point Likert scale ranging from ‘never’ to ‘(almost) always’. Total scores are calculated by transforming the item scores into a T score ranging from 0 to 100 which has a mean of 50 and standard deviation (SD) of 10 in the original calibration sample [21], where higher scores thus signify more internalizing problems. The official item parameters were used in the CAT algorithm and T score calculations, as by PROMIS convention. Previous research has shown that the PROMIS item banks provide valid and reliable measures in Dutch children [17, 22, 23].

## Data analysis

First, within each cohort, we performed independent t tests to assess differences in mean levels of internalizing problems between the independent samples at each measurement (pre-pandemic, pandemic 1, pandemic 2), and calculated hedge’s *g* effect sizes. Second, within each cohort, proportions of children with heightened symptoms were compared between measurements (pre-pandemic, pandemic 1, pandemic 2) by performing chi-square tests.

To determine proportions of children with ‘elevated’ symptoms, based on parent reports, scores on the BPM were converted into T-scores based on the large-scale pre-pandemic population-based data of the NTR. Specifically, this norm sample (*N* = 34,038) consisted of the most recent pre-pandemic assessment of those individuals from the NTR from whom no data during the pandemic were available, thereby yielding a population representative independent sample. Detailed information about the norm sample can be found in the supplementary materials. Separate T scores were calculated depending on age (8–11 years old /12–18 years old), sex (boys/girls), and rater (mother/father). In accordance with the manual of the BPM [18], T scores < 65 were interpreted as ‘normal’ and T score > 65 as elevated.

To determine proportions of children with ‘normal’, ‘mild’ or ‘severe’ symptoms based on child reports, scores on the PROMIS scales were converted into percentiles based on previously defined cut-off scores in a representative Dutch general population sample measured before the pandemic [23, 24]. The cut-off from normal to mild symptoms/function was the 75th percentile and the cut-off from mild to severe was the 95th percentile.

## Results

**Fig. 2:**
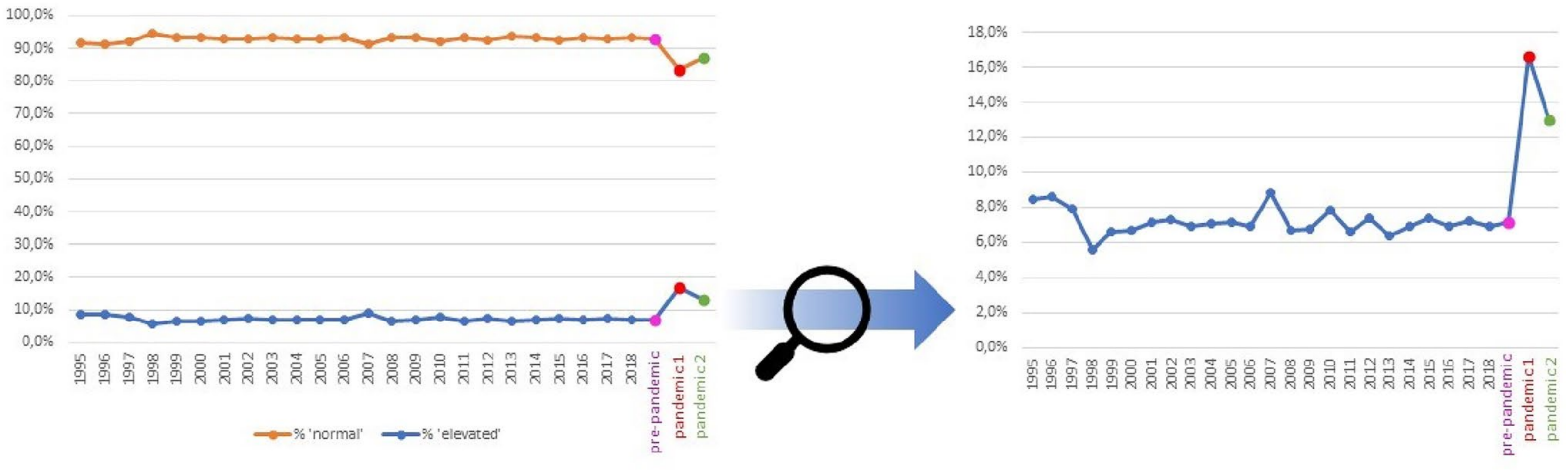
Yearly proportions of children with normal and elevated internalizing problems based on parent reports in the NTR cohort (general population) since 1995 until 2019, first pandemic measurement (Apr–May 2020), and second pandemic measurement (Nov–Dec 2020). Proportions of normal and elevated internalizing problems (left) and only elevated problems, scaled larger (right)

Table 2 displays mean scores on internalizing problems before and during the pandemic in each cohort. Table 3 displays the proportions of children with elevated internalizing problems, based on parent reports, and proportions of children with ‘normal’, ‘mild’ and ‘severe’ symptoms based on child reports, at all measurements. Figure 2 displays yearly proportions of children with normal and elevated internalizing problems based on parent reports of the NTR cohort (general population) starting in 1995 and throughout the pandemic measurements.

**Table 2:** Means and standard deviations of internalizing problems before and during the pandemic in all cohorts

Cohort	Pre-pandemic (a)		Pandemic Apr–May 2020 (b)		Pandemic Nov–Dec 2020 (c)	
	*M*	SD	*M*	SD	*M*	SD
General population						
Parent-reported internalizing problems (BPM)						
NTR	0.90^b,c^	(1.51)	1.49^a,c^	(1.99)	1.07^a,b^	(1.71)
Child-reported anxiety problems (PROMIS)						
KLIK	43.76^b,c^	(9.87)	50.78^a,c^	(7.78)	49.61^a,b^	(8.25)
Child-reported depressive problems(PROMIS)						
KLIK	44.73^b,c^	(10.62)	49.53^a^	(8.20)	48.81^a^	(9.18)
Clinical population						
Parent-reported internalizing problems (BPM)						
DREAMS	5.24	(3.24)	4.91	(3.52)	5.04	(3.41)
LDY	3.86	(3.09)	3.75	(3.16)	3.84	(3.24)
Child-reported anxiety problems (PROMIS)						
DREAMS	–	–	51.45^c^	(8.98)	54.45^b^	(9.21)
Child-reported depressive problems(PROMIS)						
DREAMS	–	–	51.98^c^	(10.64)	55.67^b^	(10.81)

*BPM *Brief problem monitor, *PROMIS *Patient-reported outcomes measurement information system

^a,b,c^represent significant differences at *p* < .05 between measurements as indicated by independent sample *t *tests

**Table 3:** Proportions of children within subgroups based on severity of the internalizing problems before and during the pandemic in all cohorts

Cohort	Subgroup	Pre-pandemic (a)	Pandemic Apr–May 2020 (b)	Pandemic Nov–Dec 2020 (c)
General population				
Parent-reported internalizing problems (BPM)				
NTR	Elevated	7.1%^b,c^	15.6%^a,c^	10.4%^a,b^
Child-reported anxiety problems (PROMIS)				
KLIK	Normal	74.8%^b,c^	46.2%^a,c^	52.9%^a,b^
	Mild	20.0%^b,c^	46.4%^a,c^	38.3%^a,b^
	Severe	5.2%^b,c^	7.5%^a^	6.7%^a^
Child-reported depressive problems (PROMIS)				
KLIK	Normal	74.8%^b,c^	59.9%^a^	61.5%^a^
	Mild	20%^b,c^	36.9%^a^	34.0%^a^
	Severe	5.2%^b^	3.2%^a^	4.5%
Clinical population				
Parent-reported internalizing problems (BPM)				
DREAMS	Elevated	74.0%^A^	69.3%	70.9%
LDY	Elevated	49.9%	47.1%	49.0%
Child-reported anxiety problems(PROMIS)				
DREAMS	Normal	–	46.2%^c^	33.3%^b^
	Mild	–	40.0%	43.2%
	Severe	–	13.8%^c^	23.5%^b^
Child-reported depressive problems (PROMIS)				
DREAMS	Normal	–	53.2%^c^	38.7%^b^
	Mild	–	31.2%	33.2%
	Severe	–	15.6%^c^	28.1%^b^

*BPM *Brief problem monitor, *PROMIS *Patient-reported outcomes measurement information system

^a,b,c^represent significant differences at *p* < .05 between measurements within populations as indicated by *χ*^2^ test.

^A^For the pre-pandemic parent reports in the DREAMS sample the informant is unknown, therefore we excluded children with a score of 3, as they could not be categorized properly, see Table S1 for rated dependent cut-off details; remaining *N* = 1257

## General population

### Parent-reported internalizing symptoms

In the NTR general population cohort, mean levels of internalizing problems were higher during the first pandemic measurement (*M* = 1.49, *SD* = 1.99) compared to the pre-pandemic measurement (*M* = 0.90, *SD* = 1.51), *t*(37,560) =  − 19.87, *p* < 0.001, *g* = 0.38 Similarly, mean levels of internalizing problems during the second pandemic measurement (*M* = 1.07, *SD* = 1.71) were higher compared to pre-pandemic measurement, *t*(35,204) = -3.61, *p* < 0.001, *g* = 0.11. In addition, the proportions of children with elevated internalizing problems were higher during the first pandemic measurement (*X*^2^ (1, *N* = 37,562) = 316.35, *p* < 0.001) and the second pandemic measurement (*X*^2^ (1, *N* = 35,206) = 18.72, *p* < 0.001) compared to the pre-pandemic measurement.

Furthermore, mean levels of internalizing problems during the second pandemic measurement were lower compared to the first pandemic measurement, *t*(4690) = 6.44, *p* < 0.001, *g* = 0.22 and the proportion of children with elevated internalizing problems was lower during the second pandemic measurement compared to the first pandemic measurement (*X*^2^ (1, *N* = 4,692) = 19.05, *p* < 0.001).

### Child-reported internalizing symptoms

In the KLIK general population cohort, mean levels of anxiety and depressive symptoms were higher during the first pandemic measurement (*M*_*anx*_ = 50.78, *SD* = 7.68 and *M*_*dep*_ = 49.53, *SD* = 8.20) compared to the pre-pandemic measurement (*M*_*anx*_ = 43.76, *SD* = 9.87 and *M*_*dep*_ = 44.73, *SD* = 10.62), *t*_*anx*_(2149) = -18.45, *p* < 0.001, *g* = 0.77 and *t*_*dep*_(2149) =  − 11.77, *p* < 0.001, *g* = 0.49. Also, mean levels of anxiety and depressive symptoms during the second pandemic measurement (*M*_*anx*_ = 49.61, *SD* = 8.25 and *M*_*dep*_ = 48.81, *SD* = 9.18) were higher compared to the pre-pandemic measurement, *t*_*anx*_(2063) =  − 14.40, *p* < 0.001, *g* = 0.63 and *t*_*dep*_(2063) =  − 9.15, *p* < 0.001, *g* = 0.40. In addition, the proportion of children with mild and severe anxiety symptoms was higher during the first pandemic measurement (_mild:_ *X*^2^ (1, *N* = 2,151) = 168.36, *p* < 0.001; _severe:_ *X*^2^ (1, *N* = 2,151) = 4.74, *p* = 0.029) and the second pandemic measurement (_mild:_ *X*^2^ (1, *N* = 2,065) = 81.87, *p* < 0.001; _severe:_ *X*^2^ (1, *N* = 2,065) = 10.00, *p* = 0.002) compared to the pre-pandemic measurement. Also, the proportion of children with mild depressive symptoms was higher during the first pandemic measurement (_mild:_ *X*^2^ (1, *N* = 2,151) = 73.84, *p* < 0.001) and the second pandemic measurement (_mild:_ *X*^2^ (1, *N* = 2,057) = 48.79, *p* < 0.001) compared to the pre-pandemic measurement.

Furthermore, mean levels of anxiety symptoms during the second pandemic measurement were lower compared to the first pandemic measurement, *t*(1576) = 2.91, *p* = 0.004, *g* = 0.15. Also, proportions of children with severe anxiety symptoms remained the same (*p* > 0.05), proportions of children with mild anxiety symptoms were lower (*X*^2^ (1, *N* = 1,578) = 10.44, *p* = 0.001), and the proportion of children that show normal anxiety symptoms were higher (*X*^2^ (1, *N* = 1,578) = 7.27, *p* = 0.007) during the second pandemic measurement compared to the first pandemic measurement. Mean levels of depressive symptoms did not differ between the first and the second pandemic measurement (*p* > *0.05*) and no differences were found in proportions of children with severe, mild, or normal depressive symptoms from the first to the second pandemic measurement (*p* > 0.05).

## Clinical Population

### Parent-reported internalizing symptoms

In both clinical populations, no differences were found in internalizing problems between pre-pandemic measurement and pandemic measurements (*p* > 0.05) nor between the two pandemic measurements (*p* > 0.05).

### Child-reported internalizing symptoms

In the DREAMS clinical sample, mean levels of anxiety and depressive symptoms were higher in the second pandemic measurement (*M*_*anx*_ = 54.45, *SD* = 9.21 and *M*_dep =_ *M* = 55.67, *SD* = 10.81) compared to the first pandemic measurement (*M*_anx_ = 51.45, *SD* = 8.98 and *M*_dep =_ *M* = 51.98, *SD* = 10.64), *t*_*anx*_(783) = 4.39, *p* < 0.001, *g* = 0.33 and *t*_dep_(768) = 4.53, *p* < 0.001, *g* = 0.34. In addition, the proportion of children with severe anxiety and depressive symptoms was higher during the second pandemic measurement compared to the first pandemic measurement (*X*^2^_anx_ = 10.48, *p* = 0.001 and *X*^2^_dep_ = 15.17, *p* < 0.05, the proportion of children with mild symptoms remained the same (*p* > 0.05) and the group with normal symptoms was smaller during the second pandemic measurement compared to the first pandemic measurement (*X*^2^_anx_ = 12.54, *p* < 0.001 and *X*^2^_dep_ = 14.82, *p* < 0.001).

## Discussion

In this study, we assessed parent- and child-reported internalizing problems in children and adolescents aged 8 to 18 years before the first Dutch COVID-19 pandemic lockdown, during the first peak/Dutch lockdown (Apr–May 2020), and during the second peak/Dutch partial lockdown (Nov–Dec. 2020) in two general population cohorts and two clinical cohorts. In the general population, we found that internalizing problems were higher during the first peak of the pandemic compared to pre-pandemic based on both child and parent reports. Yet, over the course of the pandemic, on both child and parent reports, we observed similar or even lower levels of internalizing problems. Children in the clinical population reported higher levels of internalizing symptoms over the course of the pandemic, while parents did not report differences in internalizing symptoms from pre-pandemic to the first peak of the pandemic nor over the course of the pandemic.

Our findings in the general population, of higher levels of internalizing problems during the first peak compared to pre-pandemic, are in line with prior research [6–8, 11–13]. At the start of the first pandemic peak, both children and adults were subjected to significant changes in their psychosocial environment due to the implementation of social distancing measures. Given that social interactions are fundamental to a healthy development in children and adolescents [1, 2], the sudden social deprivation and changes in daily routines as introduced by lockdown (e.g., closure of schools and social/sports clubs) may have contributed to the observed higher levels of depressive symptoms and anxiety at the start of the pandemic, as reported in this study by both parents and children themselves. Our finding that levels of internalizing problems did not differ or were lower over the course of the pandemic is in line with another study showing that anxiety and depressive symptoms subsided in adolescents of the general population in the four months after the first peak of the pandemic [25]. Specifically, concerns about home confinement and school (e.g. transitioning to online learning) have been shown to be strongly associated with increased anxiety and depressive symptoms since the onset of the pandemic [25]. Therefore, the relaxation of home confinement measures after the first peak of the pandemic and habituation to the new online school environment may have contributed to our finding that levels of anxiety and depressive symptoms did not differ or were lower over the course of the pandemic in children and adolescents of the general population.

In the clinical population, we saw higher levels of child-reported internalizing problems over the course of the pandemic. Literature indicates that children in clinical populations overall have less resilience than children without pre-existing mental health problems [26]. Resilience represents the capacity to quickly adapt to adversity, and being less resilient has been associated with worse physical, mental and emotional functioning [27]. As such, children with pre-existing problems may experience more difficulties as the pandemic continued. Furthermore, children in clinical populations may have experienced a change in treatment quality during times of the pandemic, due to increased demands on mental health services, which may have led again to an exacerbation of their internalizing problems [28]. In contrast, parents of children from the clinical population did not report any differences in their children’s internalizing problems from pre-pandemic to the first peak of the pandemic nor over the course of the pandemic. These results could indicate that the changes in their children’s mental health (as reported by the children themselves) are less noticed by the parents of children with pre-existing problems. For example, earlier studies have shown that in families of child mental health patients, family routines and functioning are already substantially accommodated to the needs of the child [29, 30], whereby a stressful life change, such as the pandemic —from a parent’s perspective— may not have introduced changes significant enough to considerably alter their perception of their child’s functioning. Also, previous studies have shown that internalizing problems —in contrast to externalizing problems— may be less readily noticed by parents [34, 35]. This may result in greater rater discrepancies, especially in vulnerable populations. Another explanation could be that, parents of children with pre-existing problems may perceive changes in their child’s mental health as less problematic, knowing that newly arising problematics will be promptly addressed within the framework of their child’s ongoing youth/psychiatric care. However, a possible ceiling effect could also explain our results, as parent-reported internalizing problems for the clinical population were already high before the pandemic, and the parental questionnaire (BPM) may not have been sensitive enough to capture increases in internalizing problems during the pandemic.

Whereas in the clinical cohort, we saw higher levels of internalizing problems as the pandemic continued, this pattern stands in contrast to the similar or lower levels of internalizing problems we found in the general population cohorts over the course of the pandemic. Specifically, given that child mental health patients may have a different psychosocial environment than children of the general population [29], the changes in government regulations throughout the pandemic (during our Nov–Dec pandemic measurement), such as re-opening of schools and social/sports clubs, may have favorably affected children of the general population but to a lesser extent the clinical populations. For example, more contact with peers may have contributed to fewer internalizing problems for children of the general population, whereas for children of clinical populations such peer contact may at baseline be more compromised (e.g., mental health problems may interfere with psychosocial functioning) or may not represent a correlate of improved mental health (e.g., school/peer group settings may perpetuate anxiety problems). Thus, the differences in the social environment/psychosocial functioning in these two populations may have amplified divergence in internalizing problems in these two populations over the course of the pandemic.

Some limitations of the present study need to be addressed. First, child reports in the clinical cohort before the pandemic were missing, and as such no inferences can be made of how great the initial impact of the pandemic was as experienced by children in this population. Moreover, none of the samples had collected data at all measurements on both parent and child reports, and representativeness of the samples could not be checked except for the general population cohort (KLIK). Families participating in the NTR generally show high socioeconomic status [16], which may have resulted in a slight overestimation of differences between clinical and population samples, in line with literature showing that children and adolescents of families with higher socioeconomic status experienced fewer emotional and behavioral problems in stressful life situations [31]. However, since we compared internalizing problems at the various time points for each sample separately, not controlling for sociodemographic differences may only have impacted generalizability. Furthermore, the mean age of children in the pre-pandemic and especially pandemic sample of the NTR is lower (childhood age range) than the mean age of the other samples (adolescent age range). In line with literature indicating that the COVID-19 pandemic may have especially perpetuated adolescents’ internalizing problems [25, 32], the NTR sample in our present study may as such have exhibited comparably smaller differences in internalizing problems before versus during the pandemic. Lastly, the samples at the various measurements in the separate cohorts are independent, so no inferences about within-person changes in internalizing behavior over time could be made, calling for future longitudinal research to address this.

The present study also has several strengths. We included large samples with children from both the general and clinical population and collected both parent and child reports. Also, the male-to-female ratio in our clinical samples is representative of the male-to-female ratio of the total population of the four Dutch psychiatric centers that were included in this study, thereby increasing generalizability of our results. Furthermore, we were able to compare the data that were collected during the pandemic with data that were collected yearly from 1995 until 2019. These yearly measurements show that proportions of elevated internalizing problems in the general population ranged from 5.6 to 8.8% between 1995 and 2019, confirming that the proportions reached during the pandemic in the general population (13.0–16.6%) represent unusually elevated problems, rather than random fluctuations in proportions of internalizing problems (see Fig. 2).

In summary, our results show that in the general population levels of internalizing problems are higher since the start of the pandemic and that more children report elevated levels of internalizing problems and may require additional support. In the clinical sample, we found that levels of child- (but not parent-) reported internalizing problems were higher over the course of the pandemic. Overall, the findings indicate that children and adolescents from both the general and clinical population were affected negatively by the pandemic in terms of their internalizing problems. Attention is therefore warranted to investigate what long-term effects this may cause and to monitor if internalizing problems return to pre-pandemic levels or if they remain elevated post-pandemic. These insights, combined with future multi-informant and longitudinal research in children of both general and clinical populations, may provide relevant information for policy-makers and mental health prevention and intervention services in times of the COVID-19 or potential future pandemics.

## Supplementary Information

Below is the link to the electronic supplementary material.

Supplementary file1 (DOCX 25 KB)
